# Exploring the mental health education policies of Chinese college students: based on policy text analysis and PMC-Index model

**DOI:** 10.3389/fpubh.2025.1560582

**Published:** 2025-03-21

**Authors:** Erdi Yu, Pu Han, Xiao Fang

**Affiliations:** ^1^School of Management, Nanjing University of Posts and Telecommunications, Nanjing, China; ^2^Jiangsu Provincial Key Laboratory of Data Engineering and Knowledge Service, Nanjing, China; ^3^School of Computer Science, Nanjing University of Posts and Telecommunications, Nanjing, China

**Keywords:** college student mental health education, PMC-Index model, text mining, policy knowledge framework, content analysis

## Abstract

**Introduction:**

College students’ mental health education is crucial for holistic individual development and societal quality. It shapes well-rounded personalities, fosters innovation, and cultivates responsible citizens, contributing to social stability and national development. Our research aims to establish an evaluation system for college student mental health education policies based on the Policy Modeling Consistency Index (PMC-Index) model and text mining techniques, quantitatively assessing 15 representative policies.

**Method:**

This paper first retrieves policy documents through specialized policy databases and government websites, excluding policies that have already expired or become invalid. The sample selections of this research range from 2001 to 2024, including national guidelines and specific actions. Referring to the above steps of policy effectiveness evaluation, our research comprehensively analyses the implementation effectiveness of the foregoing policies regarding mental health curricula, service systems, crisis intervention, and family cooperation. In the following, based on 10 major variables, 38 sub-variables are constructed with a binary coding system to quantify the content of policy for objectivity. Then, the ROSTCM 6.0 software is used for text segmentation and word frequency statistics, meanwhile, keywords and semantic networks of core policies will be considered for analysis. Subsequently, the PMC-Index is computed based on the multi-input–output matrix and a diagram of the PMC-Surface is drawn with the help of MATLAB to express policy consistency and deficiencies in different dimensions intuitively.

**Results:**

Among the 15 policies, 11 exhibit Great Consistency (GC), and 4 fall under Acceptable Consistency (AC). Higher scores are found in Policy Function (X_6_), Policy Evaluation (X_8_), and Policy Goals (X_9_), indicating practical implementation and clear guidance. However, lower scores in Policy Nature (X_1_), Policy Timeliness (X_2_), Policy Issuing Agency (X_3_), and Policy Object (X_4_) reveal deficiencies in policy innovation, long-term planning, and target group coverage. Particularly, short-term policies lack sustainability, limiting their long-term effectiveness in supporting students’ mental health. Through PMC-Surface analysis, it is found that lower scores in timeliness and adaptability to target groups are primary factors contributing to surface depressions in the diagram, suggesting that these policies struggle to meet the diverse needs of different types of higher education institutions. The research also highlights significant areas for improvement in resource allocation, support system construction, and personalized mental health services. Some policies fail to address regional disparities, with weaker implementation in underdeveloped areas and insufficient safeguard mechanisms.

**Discussion:**

In the future, long-term planning for policy optimization is expected, in which priority would be given to localized resource allocation and close collaboration among schools, families, and society to ensure the comprehensiveness and sustainability of mental health education services. The research has identified certain strengths and weaknesses in the policies concerning mental health education for college students in China, thus providing theoretical references and specific recommendations that can be effectively implemented in higher education institutions.

## 1 Introduction

In recent years, as social competition has been gradually intensifying and the pace of life has been rapidly quickening, mental health has turned into a global hot issue. Research indicates that up to 28.4% of Chinese college students are affected by mental disorders (1, 2). The college years coincide with the crucial transitional phase in students’ psychological development, during which they shift from adolescence to adulthood. Once leaving their family environments and entering higher education institutions, students have to independently face various challenges, including but not limited to academic stress, social adaptation, financial loads, alteration in family relations, and campus interpersonal conflicts (3). The cumulative impact of these challenges can increase symptoms like depression, anxiety, and stress responses, significantly affecting students’ academic performance, quality of life, and social adaptability. In severe cases, prolonged psychological distress may lead to role malfunction, unfulfilled academic accomplishments, and even self-harm or suicide. These mental health issues not only pose risks to individuals but also have broader negative consequences for families and society at large (4). Given these challenges, mental health education during this stage is particularly essential. Beyond its immediate impact on academic success and well-being, it also influences students’ future career development and overall social adaptability. Despite growing awareness of mental health education among college students, many remain psychologically ill-prepared and lack effective coping mechanisms. Previous reviews employing systematic review and meta-analyses evaluated Chinese adolescents’ awareness of mental health knowledge; most studies found that the overall rate is lower than the targets in national mental health education plans, reflecting the deficiencies in Chinese adolescents’ mental health education and highlighting the urgent need to enhance the level (5). Correspondingly, relevant national departments have implemented practical policies to reduce students’ psychological distress and promote their overall development.

When formulating, expanding, and optimizing college student mental health education policies, scientific and rational policy evaluation is an essential step. Policy evaluation is a key stage in the process of public policy, systematically analyzing the effects of policy implementation through various theoretical frameworks and quantitative approaches (6, 7). These evaluation methods enable policymakers to examine the actual results of existing policies, forecast their future development trends, and evaluate their scientific soundness and efficacy (8, 9). Furthermore, policy evaluation is not merely a retrospective instrument but also reflects whether policies achieve their intended aims in practice and offers guidance for future decision-making (10, 11). In China, numerous studies have investigated the differences between the implementation effects and intended goals of college student mental health education policies. Nevertheless, most of the current research remains at the macro level, especially lacking a detailed analysis of the strengths and weaknesses of individual policies. This paper endeavors to conduct an in-depth exploration from the perspective of policy evaluation by constructing a quantitative evaluation framework to present a benchmark for perfecting the mental health education policies directed at college students.

The core objective of our research is to comprehensively assess the quality and design of existing college student mental health education policies using quantitative methods and provide constructive suggestions for future policy optimization. The main contributions of this study are as follows: (1) Our research employs text mining techniques to analyze relevant policies, extracting common characteristics and key content for a deeper and more comprehensive understanding of policy texts. (2) The research applies the PMC-Index model to empirically evaluate 15 representative policy samples. This model systematically examines internal policy consistency across multiple dimensions to assess the strengths and weaknesses of each policy. (3) By combining text mining techniques with the PMC-Index model, our research constructs a scientifically grounded quantitative evaluation framework, providing a basis for the evaluation and analysis of college student mental health education policies. (4) Through the quantitative evaluation of 15 policies, our research identifies key issues in policy design and differences in implementation effects, offering empirical evidence for policy optimization. By establishing a clear evaluation framework and conducting empirical data analysis, this research provides both theoretical support for policymakers and practical references for research and practice in related fields. Consequently, it aims to facilitate the continuous improvement of China’s college student mental health education policy system and promote the effective implementation of these policies.

The rest of our research is structured as follows: In Section 2, we review the progress of related research works on mental health education policy for college students, and discuss the methods of policy evaluation. Section 3 describes the design of our research, which encompasses text mining as well as the construction of the PMC-Index model. Section 4 shows the results of the empirical evaluation and discusses the PMC-Index scores of different policies. Section 5 summarizes the findings. Section 6 puts forward suggestions for prospective research directions and policy proposals.

## 2 Literature review

### 2.1 Research on college student mental health education policy

#### 2.1.1 Related works

Globally, with rapid societal changes, mental health education for college students and related policy research have shown a trend of rapid development. Since 2000, the number of studies on student mental health has increased significantly. Early research primarily focused on a few developed countries, such as the United States, the United Kingdom, and South Korea. As the mental health issues of college students became increasingly prominent, the volume of global research publications surged after 2015. The outbreak of COVID-19 in 2020 created a new growth point in this research field, with numerous studies focusing on the influence of the epidemic on college students’ mental health, embracing a broad spectrum of subjects such as mental health services, depression, anxiety, and academic pressure (12). In the field of college student mental health research, U.S. scholars have contributed more than 62% of the related literature, while China and South Korea also played important roles (13). Among them, the University of Michigan in the U.S. has made the most significant contributions to college mental health research, publishing nearly 100 highly influential research papers, with a citation rate far exceeding other institutions (14). Moreover, scholars such as Daniel Eisenberg have made significant contributions to this field, focusing on topics like mental health services and students’ help-seeking behavior, providing critical theoretical support for the formulation of college mental health policies (15).

Although the advanced education environment creates a favorable platform for furthering mental health, research shows that mental health problems among college students in many countries have yet to be effectively addressed, and the proportion of students receiving mental health services remains low (16). This is partly due to cultural differences, societal perceptions, and inadequate mental health service systems (17–19). Even in developed countries, the stigma associated with mental health problems remains widespread, causing many students to seek help (20). In some developing countries, economic constraints further limit the provision and accessibility of mental health services. In the future, as the global focus on college student mental health issues deepens, research and policy development will continue to move toward more diversified, systematic, and technologically driven directions. The new mental health challenges brought about by the post-pandemic era, combined with the widespread application of information technology, will push mental health research to more comprehensive dimensions, encompassing broader social support systems, community services, and personalized interventions based on big data (21). In the future, the new direction of college student mental health research should focus on how to integrate mental health education into daily teaching activities through policy adjustment and improve the accessibility and availability of mental health services, so as to more effectively tackle the difficulties brought by the global educational environment to students’ mental health.

Currently, the formulation of mental health education policies for college students in China is receiving increasing attention, with research in this area gradually becoming more comprehensive and multi-dimensional. Existing studies primarily focus on the content of policy texts and the implementation of these policies.

1. Research on the content of college mental health education policies. Existing studies have systematically analyzed the evolution of college mental health education policies in China since the reform and opening-up, employing qualitative and quantitative methods to uncover historical development patterns, policy structure optimization, and the shift from education to integrated mental health services (22–26). These studies not only cover the characteristics and changes of various stages of college mental health education policies, but also provide historical insights and theoretical foundations for policy formulation and improvement.
2. Research on the implementation of college mental health education policies. Zhang and Chen evaluated the effectiveness of freshman mental health courses in Jiangsu Province College by analyzing changes in students’ mental health levels before and after the courses, finding a positive impact on improving the psychological well-being of freshmen (27). While studies like this offer empirical support for optimizing policy implementation, they often lack detailed analysis of policy content and implementation mechanisms.

In summary, Future research should focus on systematic analysis of policy texts to clarify objectives, content, and outcomes. It should also incorporate policy tools theory to examine policy formulation and implementation processes from diverse perspectives. Additionally, expanding the research scope and establishing a unified theoretical framework will provide stronger support for optimizing college mental health education policies. This will provide both theoretical and practical guidance for improving mental health education and ensuring its sustainable development.

#### 2.1.2 The development history of college student mental health education policy

**Figure 1:**
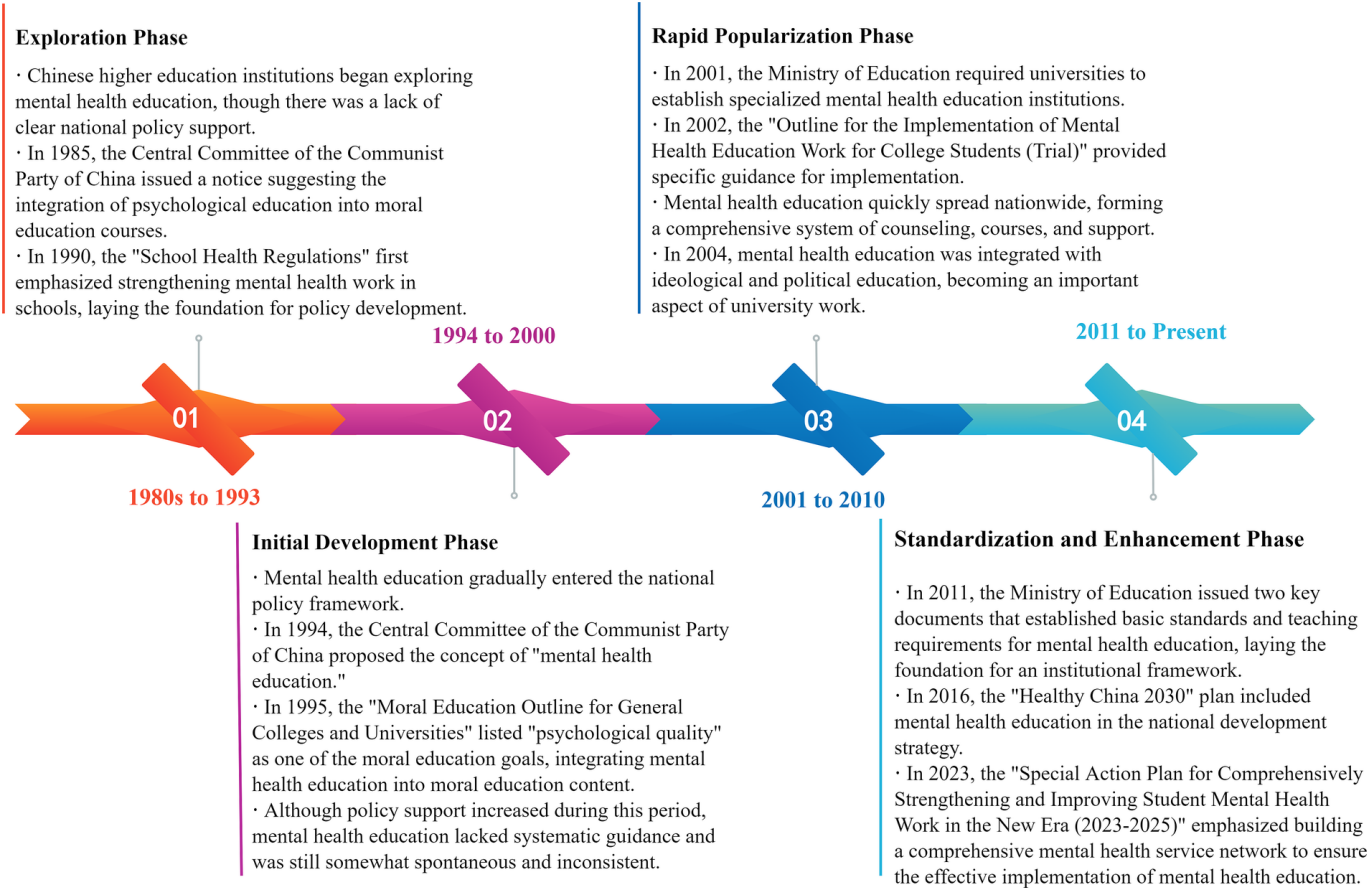
Overview of college student mental health education policy.

Since the reform and opening up, China’s college mental health education policies have experienced significant development gradually forming a systematic and standardized educational framework. As shown in Figure 1, the developmental process can be roughly segmented into four phases: the exploration phase, the initial development phase, the rapid popularization phase, and the standardization and enhancement phase. This process is shaped by the combined influence of policy promotion, societal demand, and college practices. It reflects the government’s growing emphasis on college students’ mental health issues and illustrates the gradual evolution of college mental health education policies from weakness to strength (28).

##### 2.1.2.1 Exploration phase (1980s to 1993)

In the early days of the reform and opening-up, China’s college mental health education was still in the exploratory phase, receiving limited attention. During this period, mental health services and psychological education were mainly carried out by a few colleges through voluntary counseling services and pilot courses in mental hygiene. In 1984, Beijing Normal University established the country’s first psychological measurement and counseling service center, marking the emergence of mental health education in Chinese higher education institutions (26). In 1985, the *Notice of the Central Committee of the Communist Party of China on the Reform of Ideological and Political Theory Course Teaching in Schools* explicitly mentioned the incorporation of psychological education elements into moral education, signaling the entry of mental health education into the national education policy agenda. In 1987, Zhejiang University offered the first college course on mental hygiene, initiating practical exploration of mental health education. Although early mental health education was limited in scale and impact, these initial efforts provided valuable experience for shaping future policies. In 1990, the State Education Commission issued the *Regulations on School Health Work*, which, for the first time, included provisions for strengthening psychological health work in schools, laying the policy foundation for mental health education.

##### 2.1.2.2 Initial development phase (1994–2000)

In the mid-90s, national attention to college students’ mental health gradually increased, marking the beginning of the initial development stage of mental health education policies. In 1994, the *Several Opinions on Further Strengthening and Improving School Moral Education Work*, issued by the government of China, formally brought in the notion of “mental health education.” Subsequently, the Moral Education Outline for General Colleges issued by the State Education Commission in 1995 further listed “good psychological qualities” as one of the objectives of moral education and included mental health education as a component of moral education. This marked the beginning of mental health education, which became an important part of college moral education. Although there were policy advancements during this stage, implementation remained uneven. Mental health education activities in many colleges were mainly driven by a few enthusiastic teachers and lacked unified guidance, showing spontaneity and randomness. Meanwhile, the marginalization of mental health education still exists, and many colleges do not take mental health education as a core educational task.

##### 2.1.2.3 Rapid popularization phase (2001–2010)

In the 21st century, China began to systematically promote mental health education in colleges, marking the rapid popularization stage at the national level. In 2001, the Ministry of Education issued the *Opinions of the Ministry of Education on Strengthening the Mental Health Education of College Students in Regular Institutions of Higher Learning*, which was the first policy document in China specifically targeting mental health education for college students. In 2002, the Ministry of Education further issued the *Implementation Outline for Mental Health Education of College Students in Regular Institutions of Higher Learning*, providing additional guidance and standardization on how colleges should implement mental health education. Subsequently, mental health education became a key component of college operations, with many colleges setting up mental health education centers, offering mental health courses, and hiring professional psychological counselors, thereby establishing a relatively comprehensive educational system. In 2004, the *Opinions on Further Strengthening and Improving the Ideological and Political Education of College Students* tightly integrated mental health education with ideological and political education, further advancing the institutionalization and standardization of mental health education. Under the promotion of the policy, mental health education was rapidly popularized in colleges across the country. Colleges began to regularly carry out mental health surveys, psychological lectures, group counseling, and other activities to help students improve their psychological quality.

##### 2.1.2.4 Standardization and enhancement phase (2011–present)

In 2010, mental health education in Chinese colleges entered the standardization and enhancement stage, with policy efforts gradually shifting from quantitative expansion to qualitative improvement. In 2011, the Ministry of Education issued two key policies: the *Basic Construction Standards for Mental Health Education of Students in Regular Institutions of Higher Learning* and the *Basic Requirements for Mental Health Education Course Teaching of Students in Regular Institutions of Higher Learning*. These policies set clear requirements for the construction of mental health education systems in colleges, marking the entry of mental health education into a more professionalized and standardized phase. In 2012, the state passed the *Mental Health Law of the People’s Republic of China*, further clarifying the duties and requirements for mental health education from a legal perspective, thus providing legal protection for mental health education. Since then, mental health education has been elevated to the level of national strategy. The policies from this period emphasize the professionalization and systematization of mental health education, highlighting its significance in fostering the comprehensive development of students. In 2023, the *Special Action Plan for Comprehensively Strengthening and Improving Student Mental Health Work in the New Era (2023–2025)* further proposed building a robust mental health service network, promoting the deep integration of college mental health education with the broader social psychological service system, and ensuring that mental health education would reach every student.

Overall, the development of college mental health education policies in China has been a gradual process, moving from exploratory introduction to progressive standardization, encompassing multiple stages of policy guidance, system improvement, and professional enhancement. Through continuous policy promotion, mental health education has evolved from a marginal activity to a core task in colleges, eventually becoming a part of the national strategy (22). In the future, with the development of society and the change of students’ needs, the mental health education policy of colleges at the national level will be further developed in a systematic and professional direction, which will be coordinated with ideological and political education and social psychological service system to provide strong support for students’ all-round development (24).

### 2.2 Research on policy evaluation

Policy evaluation of college mental health education is an elaborate systematic process aimed at measuring and assessing the effectiveness of policies through scientific methods. The final goal is to provide evidence to support the formulation, adjustment, and optimization of these policies (29). Therefore, choosing an appropriate policy evaluation method is important. Traditional evaluation approaches like the Analytic Hierarchy Process (AHP), BP Neural Network Model, content analysis, and social network analysis have been widely utilized in numerous fields. However, these methods have restrictions when applied to multi-criteria and intricate policies. For instance, the AHP method frequently over-simplifies complex policy structures, rendering it inappropriate for evaluating multi-criteria policies (30, 31). Meanwhile, the BP Neural Network Model has problems such as slow convergence, overfitting, and local minimization, which impact the accuracy of policy evaluation (32). Besides, content analysis can introduce subjectivity during text quantification, resulting in higher error rates (33, 34).

On the contrary, the PMC-Index model provides a more objective and systematic quantitative instrument for policy evaluation. Proposed by Ruiz Estrada, the PMC-Index model is based on the Omnia Mobilis hypothesis, which underlines that all factors in policy modeling are dynamic and interrelated (8). To thoroughly evaluate these interconnected policy factors, a multi-dimensional evaluation index system is constructed using text mining techniques (35). The PMC-Index model effectively decreases subjectivity and improves the precision and consistency of policy evaluations. In recent years, the PMC-Index model has been widely employed in various areas, including economic development, technological innovation, and social security policy evaluations (36–38). Its merit lies in its capacity to quantify the internal coherence of policy texts, analyze the coordination among different policies, and spot strengths and weaknesses in specific policy designs, thereby furnishing theoretical support for policy optimization and adjustment.

The PMC-Index model offers a new evaluation framework for college mental health education policies. The model can deeply analyze the policy text through text mining technology, identify the common features and specific needs in policy design, and reveal the consistency and difference in policy implementation. For the recent college mental health education policies released in China, multi-dimensional analysis with the PMC-Index model enables more precise evaluations of the policies’ actual effects and deficiencies in meeting students’ psychological needs and enhancing mental health. The model is cost-effective, easy to use, and significantly improves the scientific rigor and efficiency of policy evaluations. It enables researchers and policymakers to better understand policy balance, identify blind spots in design, and optimize policy content to strengthen support for students’ mental health (35).

## 3 Materials and methods

### 3.1 Data collection

This research adopted three search channels to acquire a systematic and comprehensive sample of mental health education policies for college students. First, we used the keyword “mental health education for college students” to search in specialized policy databases including the *Chinalawinfo Pkulaw Database*^1^ and the *China Law Info*.^2^ Second, we searched for policy documents related to mental health in higher education on the portals of the Chinese Ministry of Education and other relevant government websites. Finally, we supplemented the collected policies using search engines including Baidu and Google. During the document selection process, we apply the following principles: (1) primarily selecting normative policies related to college student mental health education at the national level, such as opinions, notices, implementation outlines, and action plans; (2) excluding policy documents that are no longer effective or have been revised; and (3) for policy documents only partially related to college mental health education, we select and summarize only the articles with high relevance.

Through online searches and manual reading, our research selects 15 representative policy texts closely related to college student mental health education. These policies are issued either individually or jointly by several departments, including the General Office of the Ministry of Education, the National Health Commission, the Central Publicity Department, the Central Committee of the Communist Youth League, the Ministry of Finance, the Publicity Department, the Central Civilization Office, and the Central Internet Information Office, among others (Table 1).

**Table 1:** Representative college student mental health education policies.

Item	Policy name	Issuing agency	Date issued
P1	Opinions of the Ministry of Education on Strengthening the Mental Health Education of College Students in Regular Institutions of Higher Learning	Ministry of Education	2001
P2	Implementation Outline for Mental Health Education of College Students in Regular Institutions of Higher Learning	Ministry of Education	2002
P3	Notice from the Office of the Ministry of Education on Further Strengthening the Management of College Students and Mental Health Education	Ministry of Education	2003
P4	Opinions of the Ministry of Education, Ministry of Health, and the Central Committee of the Communist Youth League on Further Strengthening and Improving the Mental Health Education of College Students	Ministry of Education, Ministry of Health, Central Committee of the Communist Youth League	2005
P5	Notice from the Office of the Ministry of Education on the Establishment of the Expert Guidance Committee for Mental Health Education of Students in Regular Institutions of Higher Learning	Ministry of Education	2005
P6	Basic Construction Standards for Mental Health Education of Students in Regular Institutions of Higher Learning (Trial)	Ministry of Education	2011
P7	Basic Requirements for Mental Health Education Course Teaching of Students in Regular Institutions of Higher Learning	Ministry of Education	2011
P8	Guiding Outline for Mental Health Education of College Students	Ministry of Education	2017
P9	Notice on the Issuance of the Pilot Work Plan for the Construction of the National Social Psychological Service System	National Health Commission, Central Political and Legal Affairs Commission, Publicity Department, Ministry of Education, Ministry of Public Security, Ministry of Civil Affairs, Ministry of Justice, Ministry of Finance, National Letters and Calls Bureau, China Disabled Persons’ Federation	2018
P10	Notice from the Ideological and Political Work Department of the Ministry of Education on Holding the ‘May 25th Mental Health Education Month’ Activity for College Students	Department of Ideological and Political Work of the Ministry of Education	2019
P11	Notice from the Office of the Ministry of Education on Strengthening the Management of Student Mental Health	Ministry of Education	2021
P12	Healthy China Action – Mental Health Action Plan for Children and Adolescents (2019–2022)	National Health Commission, Publicity Department, Central Civilization Committee Office, Central Internet Information Office, Ministry of Education, Ministry of Civil Affairs, Ministry of Finance, National Radio and Television	2019
P13	Implementation Plan for the Prevention and Treatment of Prominent Psychological Problems among Students under the Epidemic Situation of COVID-19	Ministry of Education	2022
P14	Special Action Plan for Comprehensively Strengthening and Improving Student Mental Health Work in the New Era (2023–2025)	Ministry of Education, Supreme People’s Procuratorate, Central Publicity Department, Central Internet Information Office, Ministry of Science and Technology, Ministry of Public Security, Ministry of Civil Affairs, Ministry of Finance, National Health Commission, National Radio and Television Administration, General Administration of Sport, Chinese Academy of Sciences	2023
P15	Notice from the Office of the Ministry of Education on Carrying Out the First National Student Mental Health Publicity and Education Month Activity	Ministry of Education	2024

### 3.2 Construction of the PMC-Index model

**Figure 2:**
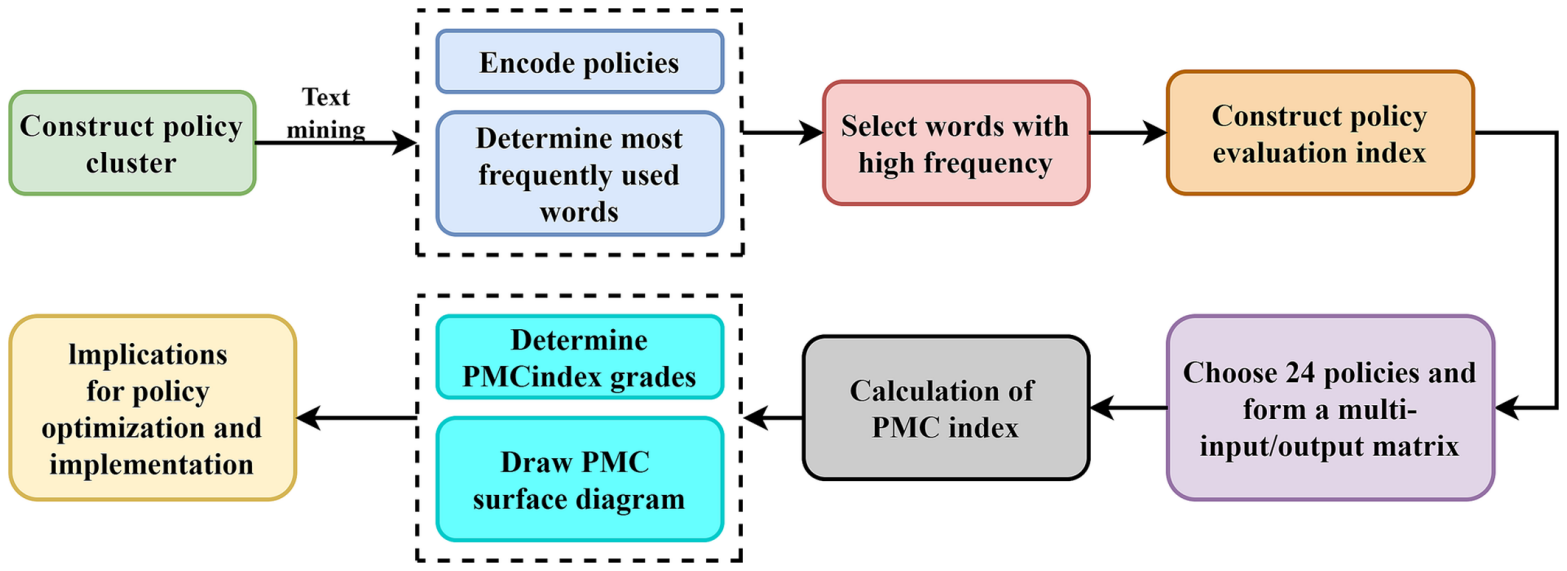
Construction framework for the PMC-Index model.

The PMC-Index model evaluates policy consistency by constructing multidimensional variables and analyzing their interrelationships. The PMC-Index model evaluates policy consistency by constructing multidimensional variables and analyzing their interrelationships (39–41). The PMC-Surface serves as a graphical representation of the model’s results, providing a visual interpretation that enables comprehensive and quantitative analysis for policy optimization. In our research, all secondary variables were assigned equal weights, and a binary method was used to balance the variables. The overall policy effectiveness was evaluated using the PMC-Index and PMC-Surface. This model can be widely applied to the quantitative analysis of different policies and provides a scientific pathway for policy improvement. The construction process of the PMC-Index model is shown in Figure 2.

#### 3.2.1 Text mining and analysis

Policy text analysis is a crucial step in identifying variables and selecting indicators for subsequent research. Our research established a text database for “college mental health education policies” and used China’s text analysis software ROSTCM 6.0 to conduct a preliminary analysis of 15 “college mental health education” policies (42). The policy texts were word segmented, then word frequency statistics were performed and high-frequency words were screened. During the statistical process, words that were irrelevant or weak in reference to this research, such as “should,” “as,” “then,” and “improve” were excluded, and the top 30 keywords by frequency were selected.

**Figure 3:**
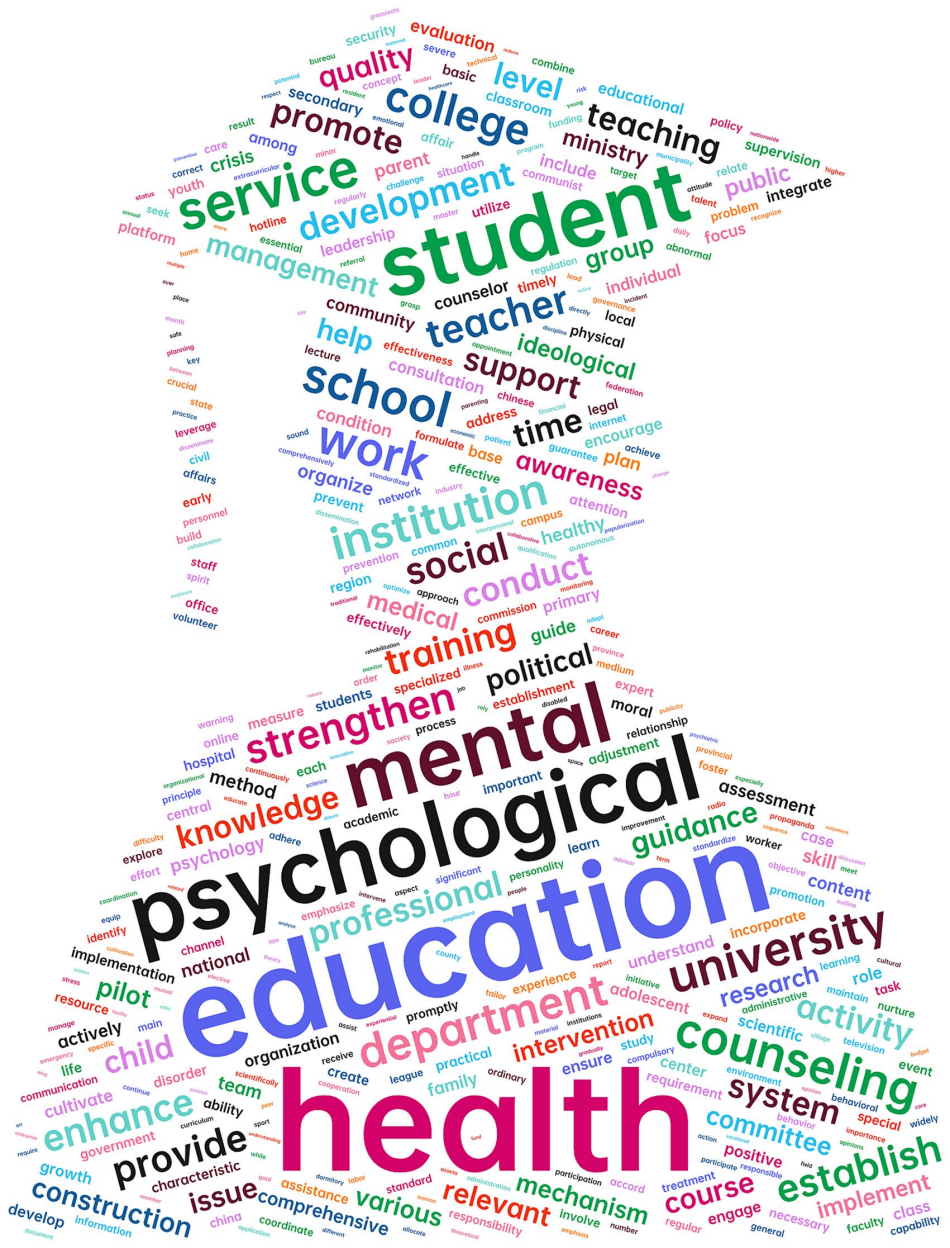
High-frequency word cloud.

As shown in Table 2, the high-frequency words in China’s “college mental health education” policies include “psychological health,” “education,” “students,” “services,” “counseling” and “strengthen” with a word cloud shown in Figure 3. These high-frequency words reflect the core content and key focus areas of the policies, indicating that the policies primarily address key themes such as “health education,” “psychological counseling,” “students,” and “schools.” The target groups of China’s “college mental health education” policies involve multiple actors, including “departments,” “schools,” “communities,” “students,” “families,” and “institution,” with particular emphasis on the two-way interaction between “students and teachers” and “schools and families.” The policy content also emphasizes the coordinated role of “mental health education” at the “school,” “society,” and “family” levels, and touches on strengthening the mental health education service system and related topics like “school intervention,” “teacher guidance,” and “psychological counseling.”

**Table 2:** High-frequency words statistics of college student mental health education.

Serial number	High-frequency words	Frequency	Serial number	High-frequency words	Frequency
1	Psychological	1,252	16	Mental	89
2	Health	826	17	Ideology	81
3	Education	596	18	Knowledge	79
4	Students	408	19	Training	77
5	Services	198	20	Guidance	75
6	Counseling	172	21	Positive	75
7	Strengthen	153	22	Organize	74
8	Teaching	142	23	Tutoring	71
9	College	117	24	Competence	67
10	Institutions	113	25	System	64
11	Departments	110	26	Capability	61
12	Teachers	104	27	Management	61
13	Construction	100	28	Intervention	60
14	Society	93	29	Publicize	60
15	Hygiene	93	30	Help	52

**Figure 4:**
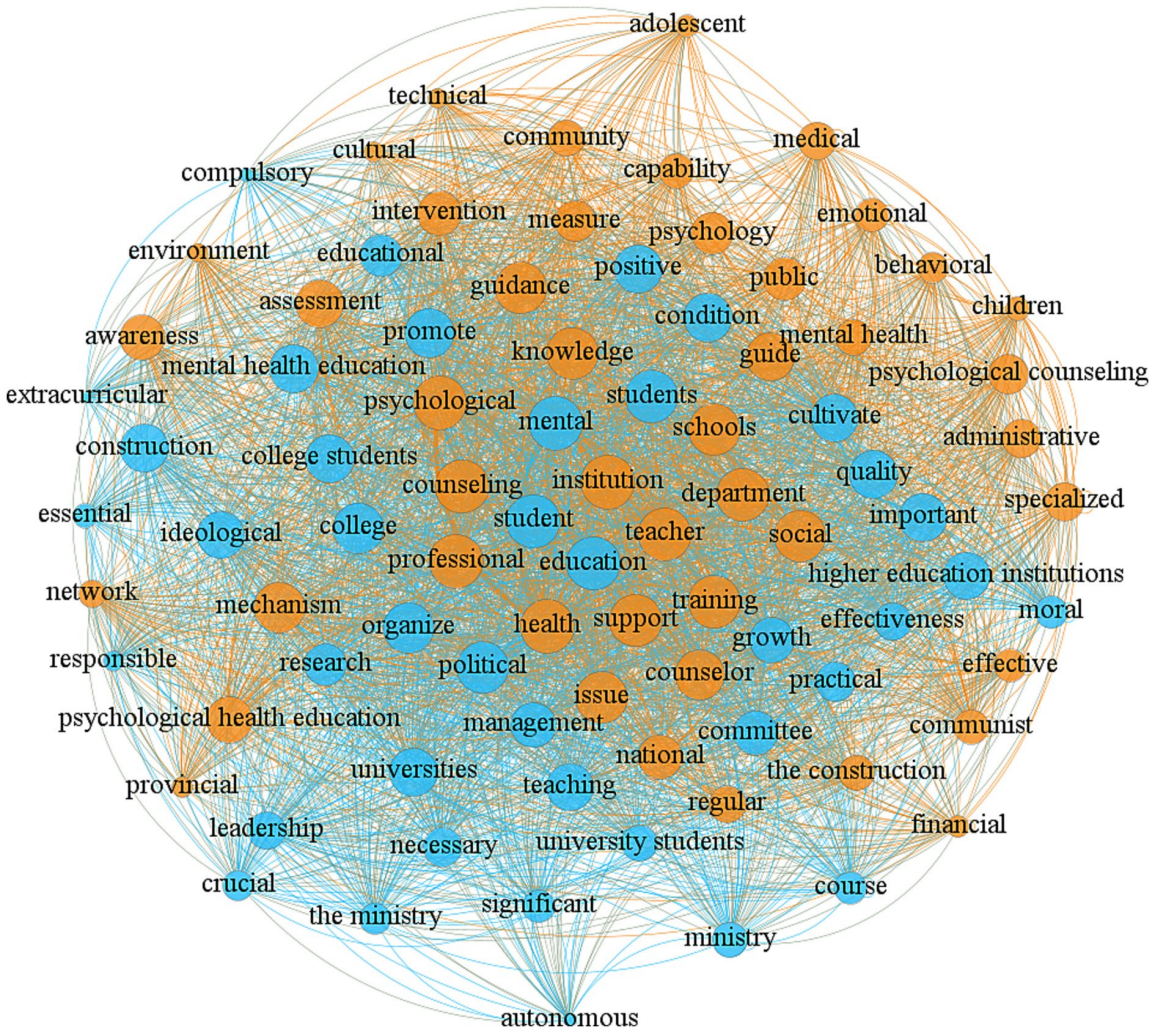
High-frequency words network diagram.

Based on the word frequency statistics, our research further handles the policies to create a co-word matrix for college mental health education policies, producing a semantic network graph of core keywords as shown in Figure 4. Each node within the network stands for a keyword, and the connections between nodes signify co-occurrence relations. The size of the nodes and the connection strength reflect the frequency and linkages of the keywords. In the semantic network graph, a node with more connections to other nodes has greater centrality, indicating its higher importance. It can be observed that central keywords like “mental,” “health,” “education,” and “college students” indicate the importance of mental health education in these policies. The analysis highlights that mental health education is not only critical to student development but also to fostering a healthy campus and promoting social harmony. Further analysis reveals that the policies cover various implementation environments, including schools, communities, and families, emphasizing a comprehensive support system. Educational models such as “teaching,” “training,” “counseling,” and “guidance” indicate a combination of theoretical education and psychological intervention. Additionally, the network graph highlights dispersed keywords such as “effectiveness,” “leadership,” “measure,” “cultivate,” “growth,” and “environment.” Although these words appear less frequently than core terms, they enrich the connotations of mental health education policies. These keywords suggest a focus on policy effectiveness, leadership in implementation, structured measures, and a supportive environment for student growth, reflecting a multi-faceted approach to mental health education.

#### 3.2.2 Variable classification and parameter identification

Based on the principles and modeling guidelines of the PMC-Index model, combined with the high-frequency words extracted from relevant policy texts and referring to the approaches of scholars such as Ruiz (35) and Zhang et al. (43, 44), this research excluded broad and insignificant indicators, ultimately identifying 10 primary variables and 38 secondary variables (45–47). The 10 primary variables are as follows: Policy Nature (X_1_), Policy Timeliness (X_2_), Policy Issuing Agency (X_3_), Policy Object (X_4_), Policy Measures (X_5_), Policy Function (X_6_), Policy Guarantee (X_7_), Policy Evaluation (X_8_), Policy Goals (X_9_), and Policy Transparency (X_10_). Through a content analysis of the collected policy texts, several sub-variables are set under each of the nine primary variables, and the complete evaluation indicator system for public data openness is presented in Table 3. Regarding Policy Transparency (X_10_), all policies are made public, so there is no need for further discussion of secondary variables in this area.

**Table 3:** Parameters setting for secondary variables.

Primary variables	Secondary variables	Meaning of secondary variables and evaluation criteria	References
Policy Nature (X_1_)	Guidance (X_1-1_)	Whether the policy is categorized as guidance: Yes, assign 1; No, assign 0	(8, 35)
	Regulation (X_1-2_)	Whether the policy is categorized as regulation: Yes, assign 1; No, assign 0	
	Notice (X_1-3_)	Whether the policy is categorized as notice: Yes, assign 1; No, assign 0	
	Opinions (X_1-4_)	Whether the policy is categorized as opinions: Yes, assign 1; No, assign 0	
	Action Plan (X_1-5_)	Whether the policy is categorized as action plan: Yes, assign 1; No, assign 0	
Policy Timeliness (X_2_)	Long-term (X_2-1_)	Whether the policy is long-term (over 5 years): Yes, assign 1; No, assign 0	(8, 35)
	Medium-term (X_2-2_)	Whether the policy is medium-term (3–5 years): Yes, assign 1; No, assign 0	
	Short-term (X_2-3_)	Whether the policy is short-term (1–3 years): Yes, assign 1; No, assign 0	
Policy Issuing Agency (X_3_)	Related to the Ministry of Education(X_3-1_)	Whether the issuing agency is related to the Ministry of Education: Yes, assign 1; No, assign 0	(51, 52)
	Multi-authority Co-issuing(X_3-2_)	Whether the policy is jointly issued by multiple authorities: Yes, assign 1; No, assign 0	
Policy Object (X_4_)	Students (X_4-1_)	Whether the policy addresses students: Yes, assign 1; No, assign 0	(53) High-frequency word statistics; Network diagram
	Schools (X_4-2_)	Whether the policy addresses schools: Yes, assign 1; No, assign 0	
	Parents (X_4-3_)	Whether the policy addresses parents: Yes, assign 1; No, assign 0	
	Society (X_4-4_)	Whether the policy addresses society: Yes, assign 1; No, assign 0	
Policy Measures (X_5_)	Curriculum Development (X_5-1_)	Whether the policy includes curriculum development: Yes, assign 1; No, assign 0	(25, 54) High-frequency word statistics; Network diagram
	Stress Management (X_5-2_)	Whether the policy includes stress management: Yes, assign 1; No, assign 0	
	Mental Health Assessment (X_5-3_)	Whether the policy includes mental health assessment: Yes, assign 1; No, assign 0	
	Early Warning and Prevention (X_5-4_)	Whether the policy includes early warning and prevention: Yes, assign 1; No, assign 0	
	Psychological Counseling and Guidance (X_5-5_)	Whether the policy includes psychological counseling and guidance: Yes, assign 1; No, assign 0	
	Home-School Collaborative Intervention (X_5-6_)	Whether the policy includes home-school collaborative intervention: Yes, assign 1; No, assign 0	
	Professional Education team building (X_5-7_)	Whether the policy includes professional education team building: Yes, assign 1; No, assign 0	
Policy Function (X_6_)	Encouragement (X_6-1_)	Whether the policy provides encouragement: Yes, assign 1; No, assign 0	(55)
	Normative guidance (X_6-2_)	Whether the policy provides normative guidance: Yes, assign 1; No, assign 0	
	Systemic restraint (X_6-3_)	Whether the policy provides systemic restraint: Yes, assign 1; No, assign 0	
	Service Optimization (X_6-4_)	Whether the policy optimizes services: Yes, assign 1; No, assign 0	
Policy Guarantee (X_7_)	Organizational Leadership (X_7-1_)	Whether the policy includes organizational leadership: Yes, assign 1; No, assign 0	High-frequency word statistics; Network diagram
	Pilot Demonstration (X_7-2_)	Whether the policy includes pilot demonstration: Yes, assign 1; No, assign 0	
	Financial support (X_7-3_)	Whether the policy includes financial support: Yes, assign 1; No, assign 0	
	Supervision and Evaluation (X_7-4_)	Whether the policy includes supervision and evaluation: Yes, assign 1; No, assign 0	
	Publicize and educate (X_7-5_)	Whether the policy includes publicizing and education: Yes, assign 1; No, assign 0	
Policy Evaluation (X_8_)	Adequate Basis (X_8-1_)	Whether the policy is evaluated based on adequate basis: Yes, assign 1; No, assign 0	(51, 56)
	Clear Objectives (X_8-2_)	Whether the policy is evaluated based on clear objectives: Yes, assign 1; No, assign 0	
	Implementation of Plans (X_8-3_)	Whether the policy is evaluated based on the implementation of plans: Yes, assign 1; No, assign 0	
	Scientific Approach (X_8-4_)	Whether the policy is evaluated based on a scientific approach: Yes, assign 1; No, assign 0	
Policy Goals (X_9_)	Enhance Psychological Health Literacy (X_9-1_)	Whether the policy aims to enhance psychological health literacy: Yes, assign 1; No, assign 0	(23, 24) High-frequency word statistics; Network diagram
	Improve Detection and Counseling Capabilities (X_9-2_)	Whether the policy aims to improve detection and counseling capabilities: Yes, assign 1; No, assign 0	
	Enhance Intervention and Disposal Capabilities (X_9-3_)	Whether the policy aims to enhance intervention and disposal capabilities: Yes, assign 1; No, assign 0	
	Strengthen Comprehensive Support Efforts (X_9-4_)	Whether the policy aims to strengthen comprehensive support efforts: Yes, assign 1; No, assign 0	
Policy Transparency (X_10_)	**/**	Whether the policy ensures transparency: Yes, assign 1; No, assign 0	(52)

#### 3.2.3 Constructing a multiple-input–output table

To quantitatively evaluate mental health education policies for college students, this study constructed a multi-input–output matrix framework. This framework can handle and store a large amount of relevant policy data. It also measures each policy variable across multiple dimensions (48, 49). According to the construction principles of the PMC-Index model, all primary and secondary variables are set with equal weights. Specifically, this approach is based on the multidimensional characteristics of policy content. We allocate values to each sub-variable by means of binary encoding. When a policy involves a specific sub-variable, we assign a value of 1; otherwise, we assign a value of 0. This approach is based on the multidimensional characteristics of policy content. Ultimately, our research identified 10 primary variables and 38 secondary variables and established the corresponding multi-input–output matrix, providing foundational data support for the subsequent calculation of the PMC-Index, as shown in Table 4 and Appendix A.

**Table 4:** Table of multi-input–output.

Primary variables	Secondary variables
X_1_	X_1-1_, X_1-2_, X_1-3_, X_1-4_, X_1-5_
X_2_	X_2-1_, X_2-2_, X_2-3_
X_3_	X_3-1_, X_3-2_
X_4_	X_4-1_, X_4-2_, X_4-3_, X_4-4_
X_5_	X_5-1_, X_5-2_, X_5-3_, X_5-4_, X_5-5_, X_5-6_, X_5-7_
X_6_	X_6-1_, X_6-2_, X_6-3_, X_6-4_
X_7_	X_7-1_, X_7-2_, X_7-3_, X_7-4_,X_7-5_
X_8_	X_8-1_, X_8-2_, X_8-3_,X_8-4_
X_9_	X_9-1_, X_9-2_, X_9-3_, X_9-4_
X_10_	/

#### 3.2.4 Calculating the PMC-Index

The PMC-Index calculation process typically involves four steps. First, the identified primary and secondary variables are input into the multi-input–output matrix, which serves as the foundation of the analysis, recording, and storing detailed data on the relationships between policy content and related variables. Second, text mining techniques are applied to assign values and perform calculations for each secondary variable based on Equations 1, 2. Third, the scores for each primary variable are calculated using Equation 3, and finally, all primary variables are combined to calculate the overall PMC-Index using Equation 4. This index measures the consistency of a policy (35).


(1) $$X∼N\left[0,1\right]$$

(2) $$X=\left\{XR:\left[0,1\right]\right\}$$

(3) $$X_{i}\left[\overset{n}{\underset{j=1}{\sum }}\frac{X_{ij}}{T\left(X_{ij}\right)}\right]$$

(4) $$\begin{array}{l} PMC-\mathrm{Index}=\overset{m}{\underset{i=1}{\sum }}\left(X_{i}\left[\overset{n}{\underset{j=1}{\sum }}\frac{X_{ij}}{T\left(X_{ij}\right)}\right]\right)=\\ \left[\begin{array}{c} X_{1}\left[\overset{5}{\underset{j=1}{\sum }}\frac{X_{1j}}{5}\right]+X_{2}\left[\overset{3}{\underset{j=1}{\sum }}\frac{X_{2j}}{3}\right]+\\ X_{3}\left[\overset{2}{\underset{j=1}{\sum }}\frac{X_{3j}}{2}\right]+X_{4}\left[\overset{4}{\underset{j=1}{\sum }}\frac{X_{4j}}{4}\right]+\\ X_{5}\left[\overset{7}{\underset{j=1}{\sum }}\frac{X_{5j}}{7}\right]+X_{6}\left[\overset{4}{\underset{j=1}{\sum }}\frac{X_{6j}}{4}\right]+\\ X_{7}\left[\overset{5}{\underset{j=1}{\sum }}\frac{X_{7j}}{5}\right]+X_{8}\left[\overset{4}{\underset{j=1}{\sum }}\frac{X_{8j}}{4}\right]+\\ X_{9}\left[\overset{4}{\underset{j=1}{\sum }}\frac{X_{9j}}{4}\right]+X_{10} \end{array}\right] \end{array}$$

Generally, the PMC-Index ranges from 0 to 9, with higher values indicating better policy consistency. For interpretative purposes, the PMC-Index can be divided into four levels, as shown in Table 5. The PMC-Index of 0–3.99 indicates poor policy consistency, 4–5.99 indicates acceptable consistency, 6–7.99 represents good consistency, and a score between 8 and 9 signifies high consistency. This classification system facilitates the quantitative analysis of overall policy effectiveness and provides a scientific basis for policy optimization and adjustment (35, 50).

**Table 5:** PMC-Index evaluation criteria.

PMC-Index	0–3.99	4–5.99	6–7.99	8–9
Evaluation	Poor consistency (PC)	Acceptable consistency (AC)	Great consistency (GC)	Perfect consistency (PC)

#### 3.2.5 Building of the PMC-Surface

To more intuitively present the PMC-Index evaluation results of college student mental health education policies, this research used MATLAB software to visualize the PMC matrix, constructing a PMC-Surface. The PMC-Surface is a 3D graphical representation of a 3 × 3 matrix composed of the nine remaining primary variables after excluding the “Policy Transparency” variable. It visually displays the evaluation results of each policy. The PMC-Surface exhibits the merits and demerits of policies in various dimensions through convex and concave patterns. The raised areas represent a higher PMC-Index, reflecting strengths, while the concave areas highlight shortcomings in related dimensions. The values of the PMC matrix are calculated by Equation 5, and the PMC-Surface is drawn accordingly, providing a clearer three-dimensional representation for policy evaluation. This visualization method effectively aids in analyzing the strengths and weaknesses of China’s college student mental health education policies and provides decision-makers with insights for policy optimization. By integrating the PMC-Surface, our research not only enables a direct assessment of the overall policy effectiveness but also offers detailed reference information for future policy adjustments and improvements. Meanwhile, through analyzing the areas with lower PMC-Index scores, targeted optimization measures can be proposed to enhance overall policy consistency and effectiveness.


(5) $$PMC-\mathrm{Surface}=\left[\begin{array}{ccc} X_{1} & X_{2} & X_{3}\\ X_{4} & X_{5} & X_{6}\\ X_{7} & X_{8} & X_{9} \end{array}\right]$$

## 4 Results and discussion

### 4.1 Evaluation object selection

This research selects 15 of the most representative national-level college student mental health education policies in China for empirical evaluation. All the selected policies are released between 2001 and 2024, covering different stages and key aspects of mental health education work. These policies involve multiple aspects, such as the establishment and teaching requirements of mental health courses, the construction and standardization of mental health service systems, psychological crisis intervention and management, student mental health assessment and intervention measures, as well as the collaborative cooperation among families, schools, and society. The core goals of the selected policies revolve around the maintenance and promotion of college students’ mental health, with a particular focus on the systematic construction of mental health education, the cultivation of professional teams, the integration of educational resources, and the supervision and evaluation of policy implementation. Some policies focus on establishing the curriculum system and standards for mental health education, while others emphasize strengthening the institutional guarantees and special action plans for mental health services. Through systematic analysis and empirical evaluation of these 15 policies, we achieve a deeper comprehension of the policy evolution and implementation effects in the field of mental health education in China, providing a powerful reference for the optimization and improvement of future policies.

These 15 national-level policies are issued by authoritative national departments and serve as the basis for the formulation of policies at the provincial level and various colleges and universities. In terms of period, these policies cover key stages of policy development, demonstrating the transformation of policies from being scattered to systematic, and from being guiding to standardized. In terms of types, they are highly diverse, including guiding documents, implementation plans, action plans, and legal frameworks. Different types of policies complement each other in function, forming a multi-level system. In addition, the issuing entities of the selected policies show strong synergy, with 11 of them jointly issued by multiple departments, reflecting their cross-field nature and having a wide-ranging influence. Finally, national-level policies possess unity, authority, and comparability, which makes them convenient for longitudinal comparison and can indirectly reflect regional differences, complementing local policies. The basic provisions and selection details of the 15 representative policies are shown in Table 1.

### 4.2 PMC-Index of 15 policy

Based on the established PMC-Index model for college mental health education policies, the research picks 15 exemplary policy texts and utilizes content analysis and text mining approaches to allocate values to the secondary variables in the multi-input–output table, creating the multi-input–output tables for these policies. According to the variable assignment results in the table, the PMC-Index of each policy is calculated, sorted, and classified. Then the PMC-Index of each policy is calculated according to the input–output table, so that the level of these 15 representative college student mental health education policies can be determined. As shown in Table 6, the average PMC-Index for these 15 policies is 6.44, and the policies are ranked in descending order as P14 > P12 > P8 > P9 > P6 > P2, P11 > P10 > P4 > P1 > P5 > P3 > P13 > P7 > P15. The top 5 policies in terms of PMC-Index are P14 (7.57), P12 (7.23), P8 (7.07), P9 (7.03), and P6 (6.78), all of which fall under the Great Consistency (GC) category. These policies perform well across multiple dimensions, especially in terms of the multiple specific sections covered by policy measures, the multi-faceted guidance of policy functions, the comprehensive implementation of policy guarantees, and the clear setting and achievement of policy goals, which effectively guide the implementation of mental health education for college students. In contrast, policies with lower PMC-Index results, such as P15 (4.68), P7 (5.40), and P13 (5.63), while performing well in some dimensions, lack comprehensive design, particularly in areas such as policy function, audience coverage, support mechanisms, and evaluation, which leaves room for improvement.

**Table 6:** The PMC-Index for 15 representative policies.

Primary variables	P1	P2	P3	P4	P5	P6	P7	P8	P9	P10	P11	P12	P13	P14	P15
X_1_	0.60	0.60	0.40	0.60	0.40	0.40	0.40	0.40	0.40	0.40	0.40	0.40	0.40	0.40	0.40
X_2_	0.67	0.67	0.67	0.67	0.67	0.67	0.67	0.67	0.33	0.33	0.67	0.33	0.67	0.67	0.33
X_3_	0.50	0.50	0.50	1.00	0.50	0.50	0.50	0.50	1.00	0.50	0.50	1.00	0.50	1.00	0.50
X_4_	0.50	0.50	0.50	0.50	0.50	0.50	0.50	0.50	1.00	0.50	0.50	1.00	0.50	1.00	1.00
X_5_	0.71	0.71	0.57	0.71	0.29	0.71	0.43	1.00	1.00	0.86	0.86	1.00	0.71	1.00	1.00
X_6_	1.00	0.75	0.75	0.75	1.00	1.00	0.75	1.00	0.75	1.00	1.00	0.75	0.75	0.75	0.50
X_7_	0.80	1.00	1.00	0.60	0.80	1.00	0.40	1.00	0.80	1.00	0.80	1.00	0.60	1.00	0.20
X_8_	0.75	1.00	0.75	0.75	1.00	1.00	0.75	1.00	0.75	1.00	1.00	0.75	0.75	0.75	0.25
X_9_	1.00	1.00	0.75	1.00	1.00	1.00	1.00	1.00	1.00	1.00	1.00	1.00	0.75	1.00	0.50
X_10_	1.00	1.00	1.00	1.00	1.00	1.00	1.00	1.00	1.00	1.00	1.00	1.00	1.00	1.00	1.00
PMC-Index	6.53	6.73	5.89	6.58	6.16	6.78	5.40	7.07	7.03	6.59	6.73	7.23	5.63	7.57	4.68
Rank	10	6	12	9	11	5	14	3	4	8	6	2	13	1	15
Level	GC	GC	AC	GC	GC	GC	AC	GC	GC	GC	GC	GC	AC	GC	AC

The results of this research show that the PMC-Index of these 15 policies ranges from 4.68 to 7.57, indicating some variability in policy consistency. According to the classification standards of the PMC-Index, the results can be categorized into Great Consistency (GC) and Acceptable Consistency (AC). Specifically, 11 policies fall into the GC category, while four policies are classified as AC, with none reaching Perfect Consistency (PC). Based on the PMC-Index calculations, the highest-ranked policy is P14, with a score of 7.57, categorized as GC. This indicates that P14 demonstrates a high level of consistency in both design and implementation, with comprehensive content and clearly defined goals, effectively covering multiple key areas and coordinating the interests of all parties. On the other hand, the lowest-ranked policy is P15, with a score of 4.68, categorized as AC. Although P15 performs well in certain dimensions, it still has room for improvement in terms of policy comprehensiveness, diversity of policy function, and audience coverage.

**Figure 5:**
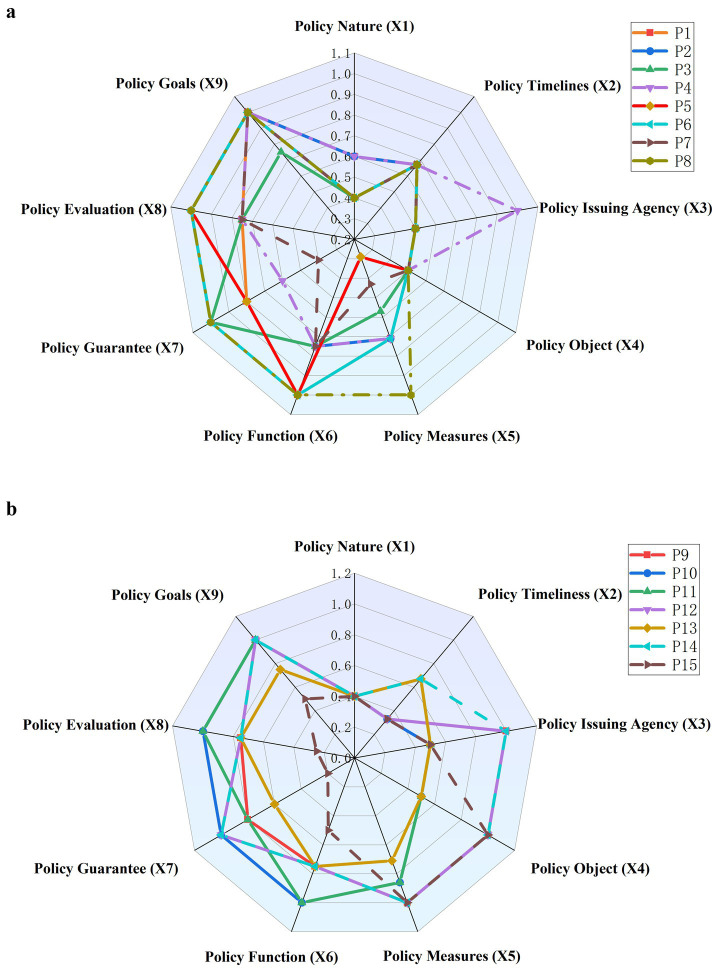
**(a)** Debra chart of the representative policies (P1–P8). **(b)** Debra chart of the representative policies (P9–P15).

To visually represent the distribution of scores for these 15 representative college student mental health education policies, our research uses Debra plots to display the scores of the main variables for each policy. As shown in Figures 5a,b, the research visualizes the scores and variations of the 15 representative policies and compares their average scores. The key findings are as follows:

1. Policy Goals (X_9_) constantly achieve high scores among the 15 policies, signifying that these policies offer clear direction and long-term planning for mental health education. Most of the policies set relatively clear goals for mental health education, which ensures that the implementation of the policy proceeds smoothly in the established direction and meets the expectations of policymakers. Except for policies P3, P13, and P15, the remaining policies all score full marks in X_9_. This implies that these policies not only set clear goals but also possess strong operability, pertinence, and adaptability. They effectively guide the implementation of policies in various colleges and provide corresponding services and support catering to the mental health needs of different groups. Such policy goals usually have wide applicability, covering diverse demographic groups and showing long-term strategic significance, permitting continuous optimization and adjustment during implementation.
2. On the whole, the average scores of Policy Measures (X_5_) and Policy Function (X_6_) are relatively high, yet there is score fluctuation. This indicates that most of the policies have a certain practical orientation at the design level, which provides specific action guidance and functional support for mental health education, but there are still fluctuations at the implementation level. It also reveals significant differences in implementation details and functional design among different policies. Policies such as P8, P9, P12, and P14 achieve e_x_cellent scores in Policy Measures (X_5_), showing that these policies offer detailed execution plans in actual operation, helping colleges effectively implement mental health education and providing tangible mental health support for students. Nevertheless, some policies have noticeably low scores in X_5_. For example, the X_5_ score of P5 is only 0.29, and that of P7 is 0.43, suggesting deficiencies in the design of specific measures. These policies either have overly simplistic operational plans lacking effective measures that meet the actual needs of colleges, or focus more on theoretical guidance without providing detailed operational instructions, resulting in difficulties in achieving the expected results during actual implementation. When promoting mental health education work, such policies fail to provide sufficient resource support or action paths, leading to insufficient influence during the implementation of policy measures. In terms of Policy Function (X_6_), policies like P8, P10, and P11 all obtain a high score of 1.00, demonstrating that these policies not only possess powerful guiding functions but also play an effective role in restraint and regulation during actual operation. Such policies usually provide a relatively comprehensive functional system, covering functional support in various aspects such as the design of mental health courses, the provision of psychological counseling services, and the establishment of crisis intervention mechanisms, ensuring the effectiveness of policy functions. However, the scores of all policies in X_6_ fluctuate between 0.75 and 1.00, suggesting that there are still differences in the actual implementation outcomes and function exertion of some policies.
3. At the same time, the scores of Policy Guarantee (X_7_) fluctuate significantly, revealing the imbalance in resource input, mechanism construction, and actual implementation capabilities within mental health education policies. The X_7_ score of P15 is only 0.20, and similarly, the X_7_ score of P7 is 0.40. Such low scores reflect severe deficiencies in providing necessary support and guarantees for these policies, severely limiting the actual effectiveness of the policies and likely leading to difficulties in generating lasting impacts during implementation. In contrast, the X_7_ scores of other policies mostly fluctuate among the values of 0.60, 0.80, and 1.00. Policies with higher scores, such as P6 and P14, provide a more comprehensive support system, covering aspects such as financial support, teacher training, and the construction of mental health facilities, thus ensuring the long-term implementation effectiveness of policies.
4. Most policies show great strength in Policy Evaluation (X_8_), suggesting well-established evaluation systems. Nevertheless, the scores of Policy Timeliness (X_2_) and Policy Nature (X_1_) are relatively low. The X_2_ scores have significant variations, especially for particular policies like P9, P10, P12, and P15. These policies only get 0.33 in X_2_, emphasizing the flaws in implementation duration and long-term effect. These limitations stem from either short policy implementation durations or their narrow focus on particular time frames or circumstances, lacking continuous planning and consistency. Furthermore, policies with low timeliness scores make it hard for policymakers to gather sufficient implementation feedback for adjustments and optimization, and they cannot respond nimbly to actual demands during execution. Although these policies may have remarkable effects at certain times, their long-term contribution to improving student mental health may fall short of expectations. In contrast, some policies have better timeliness. For instance, P6 scores 0.67 in X_2_, signifying a longer implementation cycle that provides continuous psychological support across different phases. A higher timeliness score also suggests long-term planning, meaning the policy can adapt its strategies over time to suit the changing educational environments and student needs.
5. In terms of Policy Issuing Agency (X_3_) and Policy Object (X_4_), only policies P9, P12, and P14 simultaneously achieve a high score of 1.00, suggesting excellent performance in terms of policy hierarchy and coverage of target groups. Specifically, these policies enjoy strong hierarchical support and are capable of accurately identifying and clearly addressing the mental health education requirements of diverse groups. They take into account not only students and teaching staff within schools but also external groups such as families and society, thus forming a multi-level and multi-dimensional mental health support network. Such policies are often able to mobilize the collaborative efforts of multiple parties to create a powerful policy support system. On the contrary, policies with lower scores in Policy Issuing Agency (X_3_) are often confined to the education field, lacking cross-departmental collaboration, which makes it difficult to comprehensively mobilize social resources and the power of local governments, thus limiting the scope and depth of policy implementation. Policies with lower scores in Policy Object (X_4_) tend to focus narrowly on student groups, neglecting other important stakeholders. Most of these policies are school-centered, lacking sufficient guidance for family education and social collaboration mechanisms, resulting in a lack of a comprehensive support system when dealing with complex psychological problems of students.

### 4.3 The PMC-surface of the 15 policy

This research constructs PMC matrices for 15 college student mental health education policies based on the PMC-Index, as shown in Table 7. Using these PMC matrices, PMC-Surface charts are generated for the 15 policies, detailed in Appendix B. The horizontal axis (1–3) represents the matrix’s rows, and the vertical axis (1–3) represents the columns. The variables in the matrix can be plotted using the surface chart’s coordinate system. This three-dimensional visualization allows for the intuitive observation of each policy’s performance across different stages and dimensions (57–59). Variations in color intensity on the PMC-Surface represent policy scores: darker colors indicate higher scores, while lighter colors indicate lower scores. Convex areas highlight strong performance in specific dimensions, whereas concave areas reveal potential weaknesses. Through detailed analysis of the PMC-Surface charts, we can identify each policy’s strengths and weaknesses, thereby proposing targeted optimization strategies. Based on the above analysis, the research proposes potential pathways for policy optimization.

**Table 7:** The PMC matrix of 15 policies.

Policy	P1	P2	P3	P4	P5
Policy-Matrix	$\left[\begin{array}{ccc} 0.60 & 0.67 & 0.50\\ 0.50 & 0.71 & 1.00\\ 0.80 & 0.75 & 1.00 \end{array}\right]$	$\left[\begin{array}{ccc} 0.60 & 0.67 & 0.50\\ 0.50 & 0.71 & 1.00\\ 0.75 & 1.00 & 1.00 \end{array}\right]$	$\left[\begin{array}{ccc} 0.40 & 0.67 & 0.50\\ 0.50 & 0.57 & 0.75\\ 1.00 & 0.75 & 0.75 \end{array}\right]$	$\left[\begin{array}{ccc} 0.60 & 0.67 & 1.00\\ 0.50 & 0.71 & 0.75\\ 0.60 & 0.75 & 1.00 \end{array}\right]$	$\left[\begin{array}{ccc} 0.40 & 0.67 & 0.50\\ 0.50 & 0.29 & 1.00\\ 0.80 & 1.00 & 1.00 \end{array}\right]$

Policy	P6	P7	P8	P9	P10
Policy-Matrix	$\left[\begin{array}{ccc} 0.40 & 0.67 & 0.50\\ 0.50 & 0.71 & 1.00\\ 1.00 & 1.00 & 1.00 \end{array}\right]$	$\left[\begin{array}{ccc} 0.40 & 0.67 & 0.50\\ 0.50 & 0.43 & 0.75\\ 0.40 & 0.75 & 1.00 \end{array}\right]$	$\left[\begin{array}{ccc} 0.40 & 0.67 & 0.50\\ 0.50 & 1.00 & 1.00\\ 1.00 & 1.00 & 1.00 \end{array}\right]$	$\left[\begin{array}{ccc} 0.40 & 0.33 & 0.50\\ 1.00 & 1.00 & 0.75\\ 0.80 & 0.75 & 1.00 \end{array}\right]$	$\left[\begin{array}{ccc} 0.40 & 0.33 & 0.50\\ 0.50 & 0.86 & 1.00\\ 1.00 & 1.00 & 1.00 \end{array}\right]$

Policy	P11	P12	P13	P14	P15
Policy-Matrix	$\left[\begin{array}{ccc} 0.40 & 0.67 & 0.50\\ 0.50 & 0.86 & 1.00\\ 0.80 & 1.00 & 1.00 \end{array}\right]$	$\left[\begin{array}{ccc} 0.40 & 0.33 & 0.50\\ 1.00 & 1.00 & 0.75\\ 1.00 & 0.75 & 1.00 \end{array}\right]$	$\left[\begin{array}{ccc} 0.40 & 0.67 & 0.50\\ 0.50 & 0.71 & 0.75\\ 0.60 & 0.75 & 0.75 \end{array}\right]$	$\left[\begin{array}{ccc} 0.40 & 0.67 & 0.50\\ 1.00 & 1.00 & 0.75\\ 1.00 & 0.75 & 1.00 \end{array}\right]$	$\left[\begin{array}{ccc} 0.40 & 0.33 & 0.50\\ 1.00 & 1.00 & 0.50\\ 0.20 & 0.25 & 0.50 \end{array}\right]$

**Figure 6:**
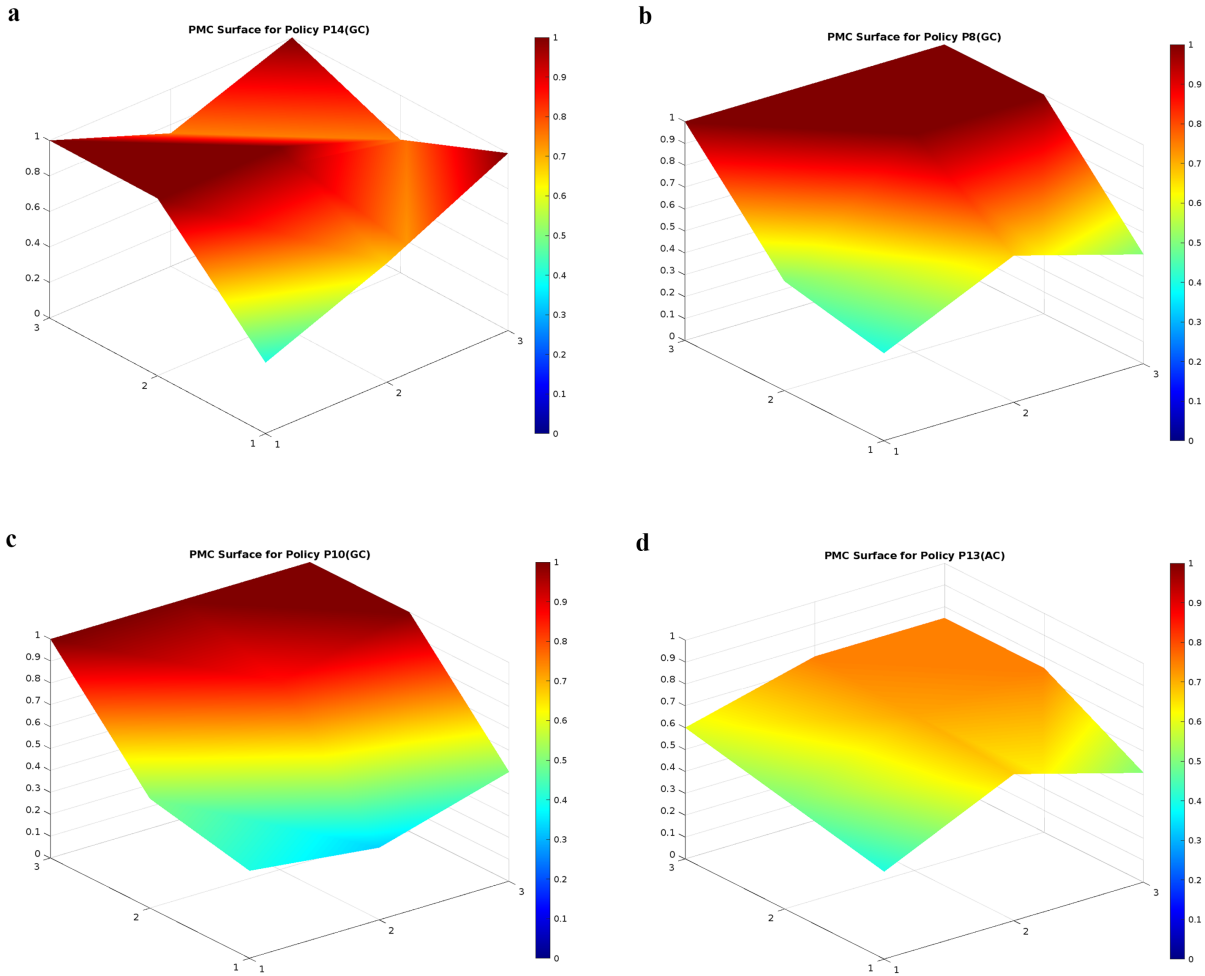
**(a)** The PMC-Surface for P14. **(b)** The PMC-Surface for P8. **(c)** The PMC-Surface for P10. **(d)** The PMC-Surface for P13.

Given the large number of samples, and to more clearly and effectively present the deficiencies and weaknesses of various college student mental health education policies, the research selects policies P14 (CG), P8 (CG), P10 (GC), and P13 (AC) as representatives from high, medium, and low levels, as shown in Figures 6a–d. These policies include different types such as educational guidelines, activity organization programs, and emergency response in special periods, covering both high-scoring policies that have been well implemented and low-scoring policies with poor execution. Through an in-depth analysis of these six policies, our research provides a comprehensive evaluation of the strengths and challenges in the implementation of college student mental health education policies, offering more targeted optimization pathways. The specific analysis results are as follows.

The PMC-Index of P14 is 7.57, ranking first and rated at the “GC” level. As a systematic national policy, the P14 policy has a relatively high overall score, especially performing outstandingly in Policy Issuing Agency (X_3_), Policy Object (X_4_), Policy Measures (X_5_), and Policy Guarantee (X_7_), resulting in an obvious protrusion on its PMC-Surface. Specifically, the P14 policy is jointly issued by several important departments, with clear policy objectives, covering a wide range of targets including students, schools and all aspects of society, reflecting its comprehensiveness and coordination. In the part of policy measures, particular attention is paid to the long-term planning and practical operation of mental health education, including aspects such as curriculum design, mental health education, monitoring, early warning and intervention, ensuring the scientific and targeted nature of the policy. In addition, the policy strengthens the implementation guarantees in multiple aspects such as organizational leadership and financial support, further enhancing its executive ability and operability. Policy Object (X_4_) is a major highlight of P14. It fully considers the roles of multiple entities including students, schools, families, and society, ensuring that the policy covers all aspects and encourages extensive social participation. Meanwhile, in terms of Policy Measures (X_5_), P14 emphasizes the all-round integration of mental health education with subject teaching, management services, family and social cooperation, especially clarifying the coverage and specific implementation plans of mental health education courses, providing detailed operation guidelines for schools, families, and society. Although P14 performs excellently in many aspects, it has a relatively low score in Policy Nature (X_1_). The main reason is that the applicable scope of the policy document is relatively narrow, which affects its adaptability in practical operation. In summary, although P14 has an excellent overall performance, the operability of the policy text still has scope for improvement. To further improve the executive ability and popularity of the policy, it is recommended to strengthen the organization and pertinence of the policy documents in future policy releases, making them more convenient for education departments at all levels and schools to implement and execute. The suggested optimization path is: X_1_–X_2_–X_6_–X_8_.

The PMC-Index of P8 is 7.07, ranking third and rated at the “GC” level. As a national guiding document for mental health education, P8 provides a detailed framework and directions for higher education institutions to implement mental health education. This policy has a wide influence in promoting mental health education in colleges across the country. It performs excellently especially in Policy Measures (X_5_), Policy Function (X_6_), and Policy Guarantee (X_7_), with scores of 1.00 respectively, reflecting the comprehensiveness of its policy measures and the high efficiency of its implementation. Specifically, P8 has made detailed provisions for the promotion of the mental health education curriculum system, the conduct of publicity activities, the optimization of psychological counseling services, and the strengthening of prevention and intervention, ensuring the orderly implementation of all aspects of work. Additionally, the policy also demonstrates powerful safeguard measures in terms of organizational leadership and financial support, forming a complete mental health education work system. However, this policy also has some drawbacks. Although it has a wide coverage, it is relatively weak in the segmentation of target groups. It also has relatively low scores in Policy Nature (X_1_) and Policy Issuing Agency (X_3_), which are 0.40 and 0.50, respectively. This may be because the issuing agency of the policy is relatively single, mainly led by the Ministry of Education, failing to form a multi-party collaborative issuing model and lacking more specific and targeted policy forms, resulting in limitations in the adaptability and implementation flexibility of the policy. To sum up, to enhance the influence of P8, it is recommended to pay more attention to the refinement and concretization of the policy text and strengthen the diversification of policy issuance. The recommended optimization path is: X_1_–X_2_–X_3_–X_4_.

The PMC-Index of P10 is 6.59, ranking eighth and rated at the “GC” evaluation level. The P10 policy takes advantage of China’s “5·25” Mental Health Education Month for College Students to intensively raise students’ mental health awareness and popularize relevant knowledge. It promotes the in-depth development of college student mental health education in the forms of diversified publicity, education, cultural and sports activities, and psychological counseling services. P10 performs well in Policy Measures (X_5_). It puts forward diversified activity forms, effectively combines psychological education with practical intervention means, promotes the improvement of students’ psychological quality in an edutainment way, and enriches the implementation paths of mental health education. P10 has a relatively high score in Policy Guarantee (X_7_), indicating that the policy has relatively complete arrangements in terms of organizational guarantee, resource support, and supervision and evaluation. However, the P10 policy has certain limitations in Policy Object (X_4_). Although the activities cover colleges across the country, they may not fully take into account the special needs of different regions and different types of colleges. In particular, some colleges in underdeveloped areas may not be able to participate in or make full use of these short-term activities due to limitations in resources and management capabilities. In addition, the score of Policy Timeliness (X_2_) of P10 is relatively low, which leads to an obvious dent on the PMC-Surface. As it is an annual centralized activity, the short-term nature of the policy limits its long-term influence in addressing students’ long-term mental health problems and dealing with complex psychological challenges. To sum up, the short-term nature, locality, and single-group targeting of P10 limit its broader effectiveness. Therefore, it is recommended to optimize Policy Nature (X_1_), Policy Timeliness (X_2_), and Policy Object (X_4_). Consider adding long-term mental health support plans, integrating some activities of the Mental Health Education Month with the daily mental health education systems of colleges, enhancing the long-term and sustainable nature of the policy, and expanding the policy’s influence and coverage. The recommended optimization path is: X_2_–X_1_–X_3_–X_4_–X_5_.

The PMC-Index of P13 is 5.63, ranking 13th and categorized under the “AC” evaluation level. The P13 policy is formulated to address the impact of the COVID-19 pandemic on students’ mental health, mainly targeting the urgent mental health needs of students during the pandemic. The objective of this policy is to relieve students’ stress and anxiety by promoting public health knowledge, strengthening students’ mental health education, and building a mental health support system. Nevertheless, the rather low PMC-Index of the policy reveals certain limitations in diverse aspects. On the PMC-Surface, these limitations are shown by its relatively smooth and unremarkable profile lacking prominent peaks. As an emergency policy, P13 is rather temporary and lacks systematic long-term planning. Although it plays a positive role during the pandemic, the lack of clear long-term implementation paths and sustainable development strategies leads to a low score in Policy Nature (X_1_). In terms of Policy Object (X_4_), P13 mainly focuses on the student group. However, the measures in the policy are not targeted enough, especially lacking cooperation between family and school, making it difficult to conduct personalized interventions and provide support for students. Besides, there are also deficiencies in Policy Guarantee (X_7_). Specifically, there is a lack of clear arrangements for financial support and long-term supervision mechanisms, which restricts the policy’s implementation and sustainability. The P13 policy puts forward some short-term psychological intervention measures for the pandemic, but these measures lack systematization, posing a challenge to the sustainability of psychological services in the post-pandemic period. The functionality of the P13 policy mainly concentrates on emergency intervention and short-term counseling, lacking a systematic long-term mental health support system, which limits the policy’s wide applicability and long-term effectiveness. In terms of Policy Evaluation (X_8_), although the policy is implemented rapidly in emergencies, it lacks a scientific and systematic evaluation mechanism for effect tracking and feedback. During the pandemic, a quick response is indeed important, but it is also necessary to conduct regular evaluations of its implementation effects to optimize intervention methods and educational content promptly. Finally, the setting of policy goals is not specific enough, and there is a lack of clear long-term strategic directions. How to integrate mental health education with regular education after the pandemic remains a weak point in the policy content. In conclusion, to enhance the overall effectiveness of P13, efforts should be made to strengthen the long-term nature of the policy, refine the policy targets, increase financial support, and establish long-term supervision mechanisms to ensure the policy’s sustainability. Additionally, the evaluation system should be improved, and a scientific feedback mechanism should be established to adjust policy measures promptly, expand the policy’s functionality, and integrate psychological education into daily teaching and management. Therefore, the recommended optimization path for P13 is: X_1_–X_3_–X_4_–X_7_–X_2_–X_5_–X_6_–X_8_–X_9_.

### 4.4 A significant correlation between policy formulation and implementation

Through the PMC-Index analysis of the 15 college student mental health education policies, it is evident that while some policies perform well in dimensions such as Policy Goals (X_9_) and Policy Measures (X_5_), demonstrating strong theoretical guidance, there are still significant fluctuations in aspects like Policy Guarantee (X_7_) and Policy Timeliness (X_2_). These discrepancies indicate that while the scientific design of a policy is essential, its actual effectiveness is ultimately determined by the implementation process. In particular, lower-ranked policies such as P15 and P13, despite performing well in certain dimensions, exhibit deficiencies in policy guarantee, resource allocation, and execution capacity, limiting their practical impact. On the other hand, high-scoring policies like P14 and P8 have achieved notable results not only due to their well-structured policy design but also because they received strong support and execution during implementation. Therefore, it can be concluded that while systematic and scientific policy design serves as the foundation, the effectiveness of the implementation process is the key factor determining whether a policy can achieve its intended outcomes. As Pressman and Wildavsky pointed out, policy implementation is the “missing link” in the policy process, as even well-designed policies may fail without effective execution (60).

Take the “5·25” Mental Health Education Month for College Students as an example. While the policy framework is well-structured and aims to enhance students’ mental health awareness through diverse activities, its implementation faces several challenges. These include insufficient attention from institutional leaders, incomplete management mechanisms, limited investment in teacher training, and misunderstandings about the objectives of mental health education. Moreover, as a short-term initiative, its long-term impact is constrained by resource disparities among different regions and colleges. This case underscores that even well-designed policies require effective execution, adequate support, and sustainable mechanisms to achieve their intended outcomes. In contrast, Peking University provides a positive example in the implementation of college student mental health education policies. The Peking University Psychological Aid Hotline, which was launched in 2019, has been in operation for 5 years. More than 100 professionals provide 24-h services and have handled over 10,000 calls. The hotline has a sound framework, and its operation is supported by a series of quality control measures. In the face of challenges such as resources, personnel, and publicity, Peking University has actively responded and achieved good results, demonstrating the importance of improving the integration mechanism and establishing a feedback mechanism, and providing valuable experience for the implementation of college mental health education policies (61).

Based on international experience, the effective implementation of policies has a positive impact on the mental health of college students. The practical cases from Brazil and Ecuador offer significant real-world references. In 2018, a Brazilian public university launched a mental health intervention program. In this program, 86 undergraduate psychology students were engaged as key facilitators to offer mental health services to 705 college students. The intervention covered aspects such as psychological screening, group counseling, individual psychotherapy, and career development guidance. Research results show that after the implementation of this program, the psychology students’ professional competencies in counseling and mental health promotion were enhanced. Meanwhile, the participating students showed positive changes in personal growth, emotional regulation, and social skills, which led to an overall improvement in their mental well-being. Similarly, in 2020, Ecuador launched a university health promotion program. In this program, 13 students were trained as “health promotion multipliers” to support 120 college students in mental health interventions. The program included health status assessments, the formulation and implementation of intervention strategies, and final outcome evaluations, ensuring a systematic and evidence-based approach to mental health promotion. Moreover, faculty members provided professional academic support, strengthening the effectiveness of the intervention measures (62). The results demonstrated significant improvements in students’ decision-making abilities, self-confidence, and interpersonal relationships, further validating the feasibility of health-promotion-based mental health interventions.

The above cases fully illustrate the importance of effective policy implementation in achieving the desired outcomes of college student mental health education. In the process of implementing mental health education policies for college students, the attention and support from the school leadership, a sound management mechanism, and sufficient investment in teacher training are all crucial. In addition, the active participation of students, the provision of diverse mental health intervention measures, and the support from professional forces are also key factors in ensuring the effectiveness of policy implementation. The significant correlation between policy formulation and implementation is evident. Effective policy implementation is essential to realize the goals set forth in policy formulation. Without effective execution, even the most well-designed policies may fail to achieve their intended objectives. On the other hand, successful implementation can demonstrate the value and effectiveness of the policies, further reinforcing the importance of both stages. Therefore, there should be a strong connection and coordination between policy formulation and implementation, ensuring that the policies are not only well-designed but also effectively executed to achieve the desired outcomes.

## 5 Conclusion

In China, an increasing volume of research has been carried out regarding college student mental health education policies. However, most existing studies remain at the macro-evaluation level, lacking in-depth analysis of the strengths and weaknesses of specific policies. This has led to unclear evaluations of policy effectiveness and hindered the development of targeted improvement measures. Therefore, this paper systematically analyzes relevant policies using text mining techniques, extracting common features and key content. It empirically evaluates 15 representative college student mental health education policies introduced since 2001 using the PMC-Index model and text mining techniques. The results show that the combination of text mining technology and PMC-Index model can systematically identify the advantages and disadvantages of Chinese college student mental health education policies, and reveal the key problems and areas that need to be improved. More specifically, the relevant conclusions of this research are as follows.

1. Most policies in college student mental health education perform outstandingly in the three dimensions of Policy Function (X_6_), Policy Evaluation (X_8_), and Policy Goals (X_9_). These policies not only have clear objectives and long-term planning but also provide specific action guidelines and evaluation mechanisms for colleges during the implementation process, indicating that the policy design is relatively excellent in terms of guidance and operability.
2. There are significant differences in the performance of policies in Policy Measures (X_5_) and Policy Guarantee (X_7_). This result reveals the imbalance in the distribution of policy resources and the resource gaps faced by some colleges in different regions during policy implementation.
3. Although most policies establish certain feedback mechanisms in Policy Evaluation (X_8_), they are relatively weak in Policy Timeliness (X_2_) and Policy Issuing Agency (X_3_). Policies with short timeliness lack long-term planning and continuity, making it difficult to form a systematic mental health support system. The singularity of the policy issuing agency also affects the effective coordination among various departments, thereby weakening the policy’s implementation ability.
4. There are notable disparities in the performance of policies regarding Policy Object (X_4_). Only a few policies achieve high marks by addressing diverse target groups. These policies promote collaboration across multiple stakeholders, forming a comprehensive mental health support network. However, most policies remain narrowly focused on schools and students, lacking sufficient guidance for family engagement and social participation. This limited scope weakens the integration of external resources and prevents the establishment of a multi-level, multi-dimensional support system for mental health education.

It is worth noting that this research has some limitations. First, while the research provides a comprehensive evaluation through a quantitative framework, it does not explore the specifics of policy implementation. The efficacy of policies hinges not merely on their design but also on the specific implementation environments of individual colleges. Second, assessment results may be influenced by sample and methodology selection, which means that not all relevant policies are fully covered. In addition, the research focuses on policy text analysis, lacks feedback from policy recipients, and may not fully reflect real-world implementation effects. Therefore, future research could integrate qualitative methods such as interviews and surveys to explore the implementation experiences and actual effects of college student mental health education policies, thus providing a more comprehensive understanding of the challenges faced by these policies. Specifically, by interviewing educators, counselors, and students, and conducting large-scale questionnaires, we can obtain first-hand data to identify the deficiencies of policies in aspects such as educational integration, service systems, and evaluation feedback. These improvements will support future policy formulation and optimization, ensuring that policies meet the mental health needs of college students.

## 6 Research outlook

Against the backdrop of the new era, the future development of college student mental health education policies must address existing shortcomings and align with national strategic needs through systematic research and refinement. Future policy research should focus on the following areas (26):

1. Promoting Integrated Construction of Mental Health Education: Policies should explore a seamless and progressive model of mental health education from primary school to college, creating individual psychological profiles for students to ensure the hierarchy, continuity, and specificity of education.
2. Strengthening Service Systems and Optimizing Service Models: Policies should enhance the construction of mental health service systems and optimize educational curricula and psychological counseling services.
3. Building a Comprehensive Mental Health Education System: Policies should focus on combining mental health education with the “Five Educations,” establish a multi-party cooperation mechanism, and construct a mental health education framework with the participation of the whole society (63). Meanwhile, a comprehensive framework for mental health education policy formulation and implementation should be formed by integrating knowledge and methods from psychology, education, and medicine.
4. Improving Evaluation Feedback and Talent Training Mechanisms: Policies should establish regular evaluation and feedback mechanisms to ensure the effective monitoring and timely optimization of policy implementation. Additionally, the cultivation of professional talent in mental health education should be strengthened, enhancing the professional skills and service quality of educators to meet high standards in mental health education (1).
5. Strengthening Policy Implementation and Supervision: Policies should focus on the effective execution of mental health education initiatives and strengthen supervision and assessment mechanisms. Regular evaluations should be conducted to identify issues and areas for improvement, enabling timely adjustments and optimizations.
6. Encouraging Collaboration with Experts and Enhancing Professional Support: Policies should promote collaboration with mental health experts, researchers, and practitioners to provide professional guidance for policy formulation and implementation. Establishing expert advisory committees and fostering partnerships with universities, medical institutions, and research centers can enhance intervention models, improve crisis response mechanisms, and ensure policies align with the latest research.

In summary, future research on college student mental health education policies should focus on systematic, targeted, and practical approaches to comprehensively improve students’ mental health and promote the in-depth and sustainable development of mental health education. Such all-encompassing policy design and implementation can effectively enhance college students’ mental health and promote the sustained development of mental health education in higher education institutions.

## Data Availability

The original contributions presented in the study are included in the article/Supplementary material, further inquiries can be directed to the corresponding author.

## Footnotes

^1^ http://www.pkulaw.com/

^2^ https://data.lawyee.net/
